# Asymptomatic left circumflex artery stenosis is associated with higher arrhythmia recurrence after persistent atrial fibrillation ablation

**DOI:** 10.3389/fcvm.2022.873135

**Published:** 2022-09-26

**Authors:** Rodrigue Garcia, Mathilde Clouard, Fabian Plank, Bruno Degand, Séverine Philibert, Gabriel Laurent, Pierre Poupin, Saliman Sakhy, Matthieu Gras, Markus Stühlinger, Nándor Szegedi, Szilvia Herczeg, Judit Simon, Harry J. G. M. Crijns, Eloi Marijon, Luc Christiaens, Charles Guenancia

**Affiliations:** ^1^Cardiology Department, University Hospital of Poitiers, Poitiers, France; ^2^Centre d’Investigation Clinique 1402, University Hospital of Poitiers, Poitiers, France; ^3^University Clinic of Internal Medicine III/Cardiology and Angiology, Medical University of Innsbruck, Innsbruck, Austria; ^4^Cardiology Department, European Hospital Georges Pompidou, Paris, France; ^5^Cardiology Department, University Hospital, Dijon, France; ^6^Division of Geriatric Medicine, Tours University Hospital, Tours, France; ^7^Heart and Vascular Center, Semmelweis University, Budapest, Hungary; ^8^School for Cardiovascular Diseases, Maastricht University Medical Centre, Maastricht, Netherlands

**Keywords:** atrial fibrillation, ablation, electrophysiology, coronary artery disease, pathophysiology, cardiac computed tomography (CCT), pulmonary vein isolation (PVI)

## Abstract

**Background:**

The pathophysiology of persistent atrial fibrillation (AF) remains unclear. While several studies have demonstrated an association between myocardial infarction and atrial fibrillation, the role of stable coronary artery disease (CAD) is still unknown. As a result, we aimed to assess the association between CAD obstruction and AF recurrence after persistent AF ablation in patients with no history of CAD.

**Materials and methods:**

This observational retrospective study included consecutive patients who underwent routine preprocedural cardiac computed tomography (CCT) before persistent AF ablation between September 2015 and June 2018 in 5 European University Hospitals. Exclusion criteria were CAD or coronary revascularization previously known or during follow-up. Obstructive CAD was defined as luminal stenosis ≥ 50%.

**Results:**

All in all, 496 patients (mean age 61.8 ± 10.0 years, 76.2% males) were included. CHA_2_DS_2_–VASc score was 0 or 1 in 225 (36.3%) patients. Obstructive CAD was present in 86 (17.4%) patients. During the follow-up (24 ± 19 months), 207 (41.7%) patients had AF recurrence. The recurrence rate was not different between patients with and without obstructive CAD (43.0% vs. 41.5%, respectively; *P* = 0.79). When considering the location of the stenosis, the recurrence rate was higher in the case of left circumflex obstruction: 56% vs. 32% at 2 years (log-rank *P* ≤ 0.01). After Cox multivariate analysis, circumflex artery obstruction (HR 2.32; 95% CI 1.36–3.98; *P* < 0.01) was independently associated with AF recurrence.

**Conclusion:**

Circumflex artery obstruction detected with CCT was independently associated with 2-fold increase in the risk of AF recurrence after persistent AF ablation. Further research is necessary to evaluate this pathophysiological relationship.

## Introduction

The number of atrial fibrillation (AF) ablation procedures is increasing tremendously worldwide (1, 2). While the mechanisms underlying paroxysmal AF are relatively well-understood, the pathophysiology of persistent AF is complex (3), with atrial substrate and fibrosis playing a crucial role in the genesis and perpetuation of the arrhythmia (4). Myocardial infarction is often associated with AF and may be one of its underlying causes (5–7). Several mechanisms regarding this association have been proposed, including heart failure, inflammation, and atrial ischemia (8–11). Nevertheless, the extent to which asymptomatic coronary artery disease (CAD) may impact AF recurrence after ablation has not been adequately investigated. Up until now, only one single-center study, with a small number of patients and short follow-up, has been published, and no association was found between CAD and arrhythmia recurrence (12).

Preprocedural cardiac computed tomography (CCT) is often performed to assess pulmonary vein anatomy, left atrial dimensions, and to exclude left atrial thrombus before AF catheter ablation (13). Moreover, it is possible to assess the presence of coronary artery stenosis (CAS) at the same time. We consequently hypothesized that coronary stenosis on CCT may be predictive of AF ablation success rate.

The purpose of the current study was to investigate the relation between asymptomatic obstructive CAD and AF recurrence after catheter ablation of persistent AF in a large-scale European multicenter cohort.

## Materials and methods

### Study population

Consecutive adult patients with symptomatic, drug-refractory persistent AF referred for catheter ablation who underwent preprocedural CCT were screened between September 2015 and June 2018 in 5 European Cardiology Department (the University Hospital of Poitiers, University Hospital of Dijon and European Hospital Georges Pompidou in France, University Hospital of Innsbruck in Austria and Heart and Vascular Center of Semmelweis University in Hungary). Patients with previously known CAD or revascularization during follow-up were excluded. The study was conducted in accordance with the Declaration of Helsinki. All the patients included in this study received an information. According to institutional policy, approval from the Institutional Review Board was not required.

### Cardiac computed tomography

Cardiac computed tomography was performed routinely 1–4 weeks prior to AF ablation using standard-of-care, site-specific protocols. The complete heart volume was acquired on a high definition CT scanner within one gantry rotation, allowing a good image quality of the coronary arteries (14, 15). CCT analysis was performed by a single reader blinded to the procedural electrophysiology result. According to the previous study published, the severity of CAD was classified in two categories: obstructive (luminal stenosis ≥ 50%) or non-obstructive (absence of CAD or luminal stenosis < 50%) (16). Localization (right coronary, circumflex, and left anterior descending artery) of the lesion was also specified.

### Atrial fibrillation ablation

Atrial fibrillation ablation procedures were performed using a cryoballoon ablation catheter (Arctic Front Advance; Medtronic, Inc, Minneapolis, MN, USA) or an open-irrigated radiofrequency ablation catheter (NaviStar, ThermoCool, or ThermoCool SmartTouch; Biosense Webster, Inc, Diamond Bar, CA, USA; Flexability, Tacticath, Tacticath SE Abbott, Mineapolis, MN, USA) using an electroanatomical mapping system (Carto 3 or EnSite Precision). Briefly, all the patients underwent standard pulmonary vein isolation and electrical isolation was confirmed by a circular multipolar electrode mapping catheter. Additional ablation lesions, such as ablation of complex fractionated atrial electrograms, or mitral, roof, and cavotricuspid lines, were performed at the discretion of the operator.

### Endpoints and data collection

The primary objective was to assess the association between obstructive CAD and AF recurrence after persistent AF ablation. Data were collected using an anonymized spread-sheet-based template. Socio-demographic and clinical characteristics were collected at the time of AF ablation. CHA_2_DS_2_–VASc score was calculated at admission (17). Before AF ablation, all the patients underwent standard two-dimensional transthoracic echocardiography to assess left ventricular ejection fraction, calculated according to the recommendations of the American Society of Echocardiography (18).

### Follow-up

Patients were followed up from the time of AF ablation for at least 1 year. All the hospitalization and consultation reports were examined. If necessary, general practitioners and referring cardiologists were contacted to provide previously missing information. Atrial fibrillation recurrences were assessed after a 3-month blanking period (19, 20), and defined as ≥ 1 AF episode recorded during a 12 lead ECG or ≥ 1 AF episode lasting ≥ 30 s documented by Holter monitoring (21). Most of patients had an ECG at 3 months, and a 24-h Holter monitoring at 6, 12, and 24 months. Furthermore, in case of symptoms patients were asked to record an ECG.

### Statistical analysis

Continuous variables were expressed as mean and SD or median and interquartile range as appropriate, and categorical variables were reported as numbers and percentages. Comparisons between groups were performed using the Student *t*-tests or the Mann–Whitney *U* tests for continuous variables as appropriate, and χ^2^ test for categorical variables. Cumulative incidence curves were built according to the presence or absence of circumflex artery obstruction using the Kaplan–Meier method and compared using a log-rank test. For analysis of the predictive value of circumflex artery obstruction, recurrences during the first 3 months after the ablation procedure (blanking period) were not taken into account (20). Multivariate Cox analysis using an entry procedure was performed on an initial model including factors known to be associated with AF recurrence (age, body mass index, hypertension, diabetes, indexed left atrial volume, left ventricular ejection fraction, ß-blockers, amiodarone, pulmonary vein isolation alone or with additional lesion set, circumflex artery obstruction). Left anterior descending artery and right coronary artery occlusion were forced into the model in order to see if the association between circumflex artery obstruction and AF recurrence changed. Analyses were performed using SPSS 22 (SPSS, Inc., Chicago, IL, USA) and SAS 9.3 (SAS Institute Inc., Cary, NC, USA) statistical software. Two-sided *p*-values of less than 0.05 were considered statistically significant.

## Results

### Baseline characteristics

Among 652 patients who had preprocedural CCT for persistent AF ablation, 156 (23.9%) were not included because of CAD or coronary revascularization, or impossible coronary assessment due to the quality of the exam. Finally, 496 patients were analyzed. Table 1 illustrates the baseline characteristics of the population. Mean age was 61.8 ± 10.0 years, and 378 (76.2%) patients were men. Mean body mass index was 29.0 ± 5.2 kg/m^2^ and 323 (65.1%) had previous hypertension. CHA_2_DS_2_–VASc score was of 0 or 1 point in 225 (36.6%) patients and 2 or 3 points in 210 (42.3%) patients. Mean left ventricular ejection fraction was 56.4 ± 11.1% and mean left atrial indexed volume was 65.4 ± 23.7 ml/m^2^. In the terms of medications, 419 (84.4%) patients were prescribed anticoagulant therapy, 327 (65.9%) ß-blockers, and 181 (36.5%) amiodarone.

**TABLE 1:** Baseline characteristics.

	Whole population *N* = 496	AF recurrence *N* = 207	No AF recurrence *N* = 289	*P*-value
Age, years	61.8 ± 10.0	62.5 ± 9.5	61.4 ± 10.3	0.24
Male	378 (76.2)	154 (74.4)	224 (77.5)	0.42
Body mass index, kg/m^2^	29.0 ± 5.2	29.3 ± 5.4	28.3 ± 5.1	0.33
Hypertension	323 (65.1)	145 (70.0)	178 (61.8)	0.05
Diabetes	69 (13.9)	24 (11.6)	45 (15.6)	0.21
Hypercholesterolemia	230 (46.4)	89 (43.0)	141 (48.8)	0.20
CHA_2_DS_2_-VASc score				0.91
0–1	225 (36.3)	92 (44.4)	133 (46.0)	
2–3	210 (42.3)	90 (43.5)	120 (41.5)	
≥4	61 (12.3)	25 (12.1)	36 (12.5)	
Left ventricular ejection fraction,%	56.4 ± 11.1	55.0 ± 11.0	57.4 ± 11.1	0.02
Medication				
NOAC	286 (57.7)	114 (55.1)	172 (59.5)	0.32
Vitamin K antagonist	133 (26.8)	64 (30.9)	69 (23.9)	0.08
ß-blockers	327 (65.9)	141 (68.1)	186 (64.4)	0.38
ACEi	139 (28.0)	57 (27.5)	82 (28.4)	0.84
Antiplatelet agents	17 (3.4)	10 (4.8)	7 (2.4)	0.15
Flecainide	34 (6.9)	10 (4.8)	24 (8.3)	0.13
Amiodarone	181 (36.5)	75 (36.2)	106 (36.7)	0.92
AF ablation energy				0.05
Radiofrequency	431 (86.9)	187 (90.3)	244 (84.4)	
Cryoballoon	65 (13.1)	20 (9.7)	45 (15.6)	
AF ablation lesion set				0.18
PVI alone	294 (59.3)	118 (57.0)	176 (60.9)	
PVI + CFAE	70 (14.1)	29 (14.0)	41 (14.2)	
PVI + lines	114 (23.0)	48 (23.2)	66 (22.8)	
PVI + CFAE + lines	18 (3.6)	12 (5.8)	6 (2.1)	

Results are expressed as mean ± SD or number (%). ACEI, angiotensin-converting enzyme inhibitor; AF, atrial fibrillation; CFAE, complex fractionated atrial electrograms; PVI, pulmonary vein isolation; NOAC, novel oral anticoagulants.

Ablation was performed with radiofrequency ablation catheter in 431 (86.9%) patients and with cryoballoon catheter in 65 (13.1%). Among them, 294 (59.3%) had pulmonary vein isolation alone and 202 (40.7%) underwent additional lesion sets.

### Prevalence and characteristics of coronary stenosis

Obstructive CAD was present in 86 (17.3%) patients (Table 2). Obstructive CAD was observed in the left anterior descending artery, right coronary artery, and circumflex artery with 76 (15.3%), 24 (4.8%), and 25 (5.0%) patients, respectively.

**TABLE 2:** Cardiac computed tomography characteristics.

	All population *N* = 496	AF recurrence *N* = 207	No AF recurrence *N* = 289	*P*-value
Left atrial indexed volume, ml/m^2^	65.4 ± 23.7	69.3 ± 26.5	62.6 ± 21.1	< 0.01
Obstructive coronary stenosis	86 (17.3)	37 (17.9)	49 (17.0)	0.79
Number of obstructed vessels				0.11
0	410 (82.7)	170 (82.1)	240 (83.0)	
1	54 (10.9)	18 (8.7)	36 (12.5)	
2	24 (4.8)	14 (6.8)	10 (3.5)	
3	8 (1.6)	5 (2.4)	3 (1.0)	

Results are expressed as mean ± SD or number (%). AF, atrial fibrillation.

### Recurrence of atrial fibrillation and coronary artery stenosis status

During the mean follow-up of 24 ± 19 months, AF recurrence occurred in 207 (41.7%) patients. AF recurrence rate was not different when considering the number of obstructed coronary arteries: 42.9% in the non-lesion group, 41.1% in the single-vessel lesion group, 41.1% in the 2-vessel lesion group, and 40.8% in the 3-vessel lesion group (*P* = 0.98).

**FIGURE 1:**
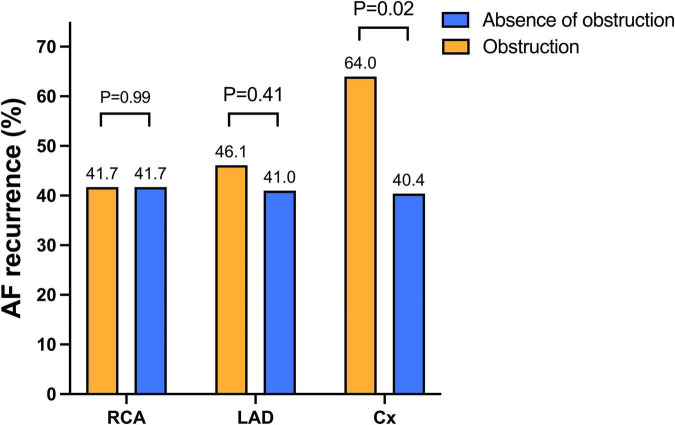
Atrial fibrillation recurrence according to the localization of the obstruction. Arrhythmia recurrences did not differ between obstructed and non-obstructed groups regarding the LAD and the RCA. On the contrary, AF recurrences were higher in patients with circumflex artery obstruction compared to patients without circumflex artery obstruction. Abbreviations: AF, atrial fibrillation; Cx, circumflex artery; LAD, left anterior descending; RCA, right coronary artery.

**FIGURE 2:**
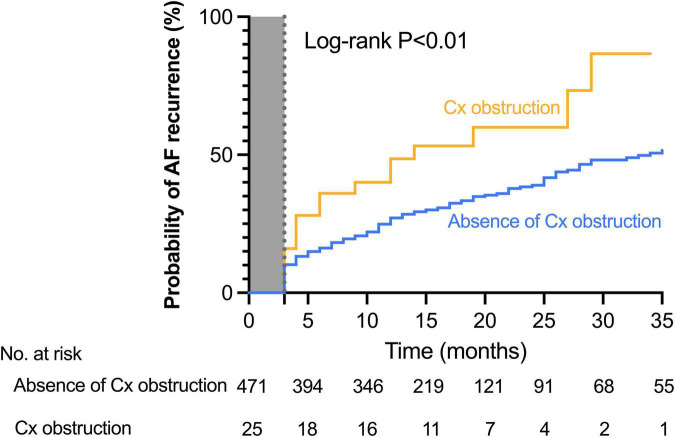
Cumulative incidence of atrial fibrillation recurrence in patients with or without circumflex coronary artery obstruction. The Kaplan–Meier estimates of the AF recurrence rate after ablation stratified by the presence or the absence of circumflex obstruction. At 2 years, documented recurrence of AF had occurred in 56.0% (14 of the 25) of patients with circumflex artery obstruction and in 32.1% (151 of the 471) of patients without circumflex artery obstruction (Log-rank hazard ratio [HR], 2.06; 95% confidence interval [CI], 1.02–4.16; *P* < 0.01). Abbreviation: Cx, circumflex artery.

In addition, AF recurrence rate did not differ between patients according to the presence of obstructive CAD [absence of obstructive CAD: 170 (41.5%) patients; obstructive CAD: 37 (43.0%) patients; *P* = 0.79]. When considering the location of the obstruction there was no difference in relation to outcome seen between those with vs. without obstruction in the left anterior descending and right coronary arteries. On the other hand, the recurrence rate was significantly higher in case of circumflex artery obstruction (Figure 1). The Kaplan–Meier analysis confirmed a higher risk of AF recurrence in the group in which circumflex artery was stenosed. At 1 year, documented recurrence of AF had occurred in 48.0% (12 out of 25) of patients with circumflex artery obstruction and in 26.1% (123 of the 471) of patients without circumflex artery obstruction. At 2 years, documented recurrence of AF had occurred in 56.0% (14 of the 25) of patients with circumflex artery obstruction and in 32.1% (151 of the 471) of patients without circumflex artery obstruction (Log-rank hazard ratio [HR], 2.06; 95% confidence interval [CI], 1.02 to 4.16; *P* < 0.01) (Figure 2). Baseline characteristics of the patients according to circumflex artery obstruction are given in Supplementary file 1.

Multivariable Cox regression analysis showed that body mass index (HR 1.04; 95% CI 1.01–1.07 per 1 kg/m^2^ increase; *P* = 0.02), left atrial indexed volume (HR 1.01; 95% CI 1.01–1.02 per 1 ml/m^2^ increase; *P* < 0.001), and circumflex artery obstruction (HR 2.32; 95% CI 1.36–3.98; *P* < 0.01) were independently associated with AF recurrences at follow-up after persistent AF ablation (Table 3). When left anterior descending artery and right coronary artery occlusion were forced in the model, those two variables were not associated with AF recurrence and circumflex artery obstruction was still associated with AF recurrences (HR 2.45; 95% CI 1.37–4.36; *P* < 0.01).

**TABLE 3:** Multivariable Cox analysis for AF recurrence after persistent AF ablation.

	Initial model			Final model		
	Hazard ratio	95% CI	*P*-value	Hazard ratio	95% CI	*P*-value
Age, per 1 year increase	1.005	0.989–1.022	0.53			
Body mass index, per 1 kg/m^2^ increase	1.035	1.006–1.065	0.02	1.033	1.005–1.062	0.02
Hypertension	1.042	0.748–1.450	0.81			
Diabetes	0.795	0.508–1.244	0.32			
ß-blockers	1.260	0.933–1.701	0.13			
Amiodarone	0.940	0.704–1.255	0.67			
LVEF, per 1% increase	0.996	0.985–1.008	0.53			
LAIV, per 1 ml/m^2^ increase	1.011	1.006–1.017	< 0.001	1.012	1.006–1.018	< 0.001
Circumflex artery obstruction	2.322	1.355–3.978	< 0.01	2.154	1.287–3.607	< 0.01
Ablation technique other than PVI combined (ref PVI only)	0.899	0.674–1.198	0.47			

LAIV, left atrial indexed volume; LVEF, left ventricular ejection fraction; PVI, pulmonary vein isolation.

## Discussion

**FIGURE 3:**
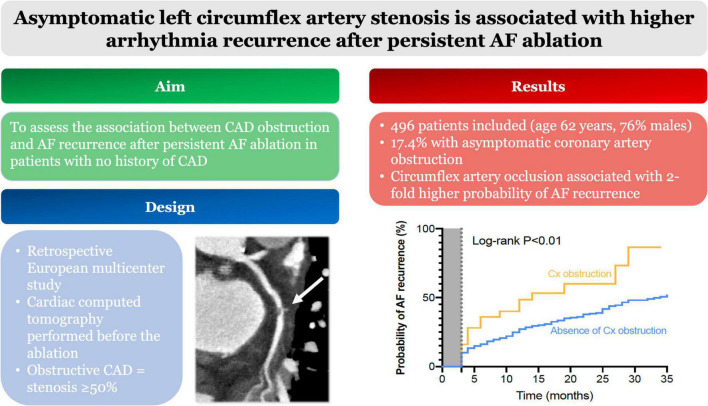
Central illustration. In total, 496 patients had CCT before persistent AF ablation. Among them 17.4% had coronary obstruction. Circumflex artery occlusion was associated with a 2-fold higher probability of AF recurrence during follow-up. Abbreviations: AF, atrial fibrillation; CCT, cardiac computed tomography.

In this large-scale international study bringing together 496 patients referred for persistent AF ablation, 5% had obstructive CAD located on the circumflex artery determined by CCT before ablation (Figure 3). This parameter was independently associated with a doubled risk of AF recurrence after ablation, along with of left atrial indexed volume and body mass index.

### Prevalence of asymptomatic coronary artery stenosis before atrial fibrillation ablation

Previous studies found prevalence of asymptomatic obstructive CAD ranging from 24 to 41% (16, 22), which was substantially higher than the proportion in our study (17.3%). This difference might be explained by the lower prevalence of cardiovascular risk factors and above all by the higher prevalence of women in our study compared to the one authored by Nucifora et al. Mito’s work had also an older population compared to ours.

### Association between coronary artery stenosis and atrial fibrillation recurrence

This is the first multicenter study assessing the impact of coronary artery stenosis on AF recurrence after persistent AF ablation. Coronary artery disease might be responsible for AF with several mechanisms: chronic inflammation, heart failure causing atrial stretch, and finally ischemia, all of which are responsible for fibrosis (4, 23–25). In our study, AF recurrence was not significantly different between patients with obstructive CAD and patients without obstructive CAD. These results are in line with a previous study carried out on a limited number of patients with shorter follow-up (12). In order to explore a possible effect of the CAD localization, Kornej et al. tried to determine whether a right coronary stenosis was associated with outcomes after AF ablation but the recurrence rate did not depend on obstruction (26). The present work is the first to evaluate the role of each coronary artery.

### Role of circumflex artery in atrial fibrillation recurrences

The anatomy of coronary arteries supplying the left atrium has been described as originating from the first segment of the circumflex artery (27, 28), and Alasady et al. demonstrated that the involvement of the left atrial coronary branch during myocardial infarction was associated with AF onset (29). Moreover, a study on experimental myocardial infarction on swine demonstrated that proximal circumflex artery occlusion involving the left atrial branch was associated with atrial infarction and atrial structural remodeling, characterized by early left atrial dilation, dysfunction, and fibrosis. When proximal circumflex artery occlusion was performed in swine without involving the left atrial branch, only interstitial atrial fibrosis was found with a lesser degree of left atrial dilation or dysfunction, and in cases of left anterior descending occlusion, no atrial fibrosis was found, and there was no left atrial remodeling (24). Taken together, these studies highlight the involvement of the circumflex artery in left atrial vascularization and suggest atrial ischemia and infarction as potential mechanisms of atrial fibrosis and AF. In the present work, only circumflex stenosis, without known clinical infarction, was associated with a higher rate of AF recurrence. These results argue for a regional and targeted, not just global (30), pathophysiological mechanism of atherosclerosis on the perpetuation of atrial fibrillation (31). The other consequence of these findings is that not only symptomatic myocardial infarction, but also silent CAD, is associated with AF recurrence. The mechanism leading to AF recurrence could consequently be silent chronic atrial ischemia related to circumflex coronary stenosis. To shed further light on these results, atrial substrate evaluation through electroanatomical mapping and cardiac magnetic resonance imaging combined with detailed coronary angiography could add new data on the role of the circumflex artery and ischemia in atrial fibrillation.

### Limitations

The absence of uniformity in CCT protocols is a methodological limitation. Nevertheless, we were able to highlight the role of circumflex artery stenosis in AF recurrence, which strengthens this result on account of the assessment heterogeneity. Second, the latest classification of coronary artery disease (CAD RAD) was not used in our study because it was not available on all CCT reports (32). However, we have chosen a cutoff (50%) that has already been used in the literature and which reflects a significant rate of CAD (16). In addition, the small number of patients with circumflex artery stenosis (25 patients with LCX obstruction vs. 471 patients without LCX) may have a significant impact on the statistics. Minor change in number recurrence might come to statistic difference. Finally, given the limited CT scan resolution for small artery assessment, we were not able to assess lesions located on the atrial branch of the circumflex artery.

## Conclusion

In a large population of patients undergoing persistent AF catheter ablation, this study demonstrates that obstructive stenosis of the circumflex artery defined by pre-procedural CT scan is independently associated with AF recurrence. Further studies are required to investigate the pathophysiology associated with this finding.

## Data availability statement

The raw data supporting the conclusions of this article will be made available by the authors, without undue reservation.

## Ethics statement

All patients included in this study received an information letter. According to institutional policy, approval from Institutional Review Board was not required. Written informed consent for participation was not required for this study in accordance with the national legislation and the institutional requirements.

## Author contributions

All authors listed have made a substantial, direct, and intellectual contribution to the work, and approved it for publication.
