# Supplementary material for: Asymptomatic left circumflex artery stenosis is associated with higher arrhythmia recurrence after persistent atrial fibrillation ablation
Source: Front Cardiovasc Med. 2022 Sep 26;9:873135. doi: 10.3389/fcvm.2022.873135 (PMC9548703; doi:10.3389/fcvm.2022.873135)
Supplement: Supplementary file 1 [file Data_Sheet_1.docx]

**SUPPLEMENTARY FILE 1** : Baseline characteristics according to left circumflex artery stenosis

|  | **Whole population** | **No LCX stenosis** | **LCX stenosis** | **P-value** |
| --- | --- | --- | --- | --- |
| Age, years | 62 ±10 | 61 ±10 | 68 ±6.3 | <0.001 |
| Male | 378 (76%) | 361 (77%) | 17 (68%) | 0.323 |
| Body mass index, kg/m^2^ | 29.0 ±5.2 | 29.0 ±5.2 | 29.4 ±4.5 | 0.567 |
| Hypertension | 323 (65%) | 303 (64%) | 20 (80%) | 0.109 |
| Hypercholesterolemia | 230 (46%) | 213 (45%) | 17 (68%) | 0.026 |
| Diabetes | 69 (14%) | 61 (13%) | 8 (32%) | 0.014 |
| CHA2DS2-VASc |  |  |  | <0.001 |
| 0-1 | 225 (45%) | 223 (47%) | 2 (8.0%) |  |
| 2-3 | 210 (42%) | 192 (41%) | 18 (72%) |  |
| ≥4 | 61 (12%) | 56 (12%) | 5 (20%) |  |
| Left ventricular éjection fraction, % | 56 ± 11 | 56 ±11 | 56 ±9 | 0.663 |
| Medication |  |  |  |  |
| NOAC | 286 (58%) | 274 (58%) | 12 (48%) | 0.316 |
| vitamine K antagonist | 133 (27%) | 125 (27%) | 8 (32%) | 0.548 |
| ß-blockers | 327 (66%) | 313 (66%) | 14 (56%) | 0.282 |
| ACEi | 139 (28%) | 135 (29%) | 4 (16%) | 0.170 |
| antiplatelet agents | 17 (3.4%) | 16 (3.4%) | 1 (4.0%) | 0.591 |
| flecainide | 34 (6.9%) | 33 (7.0%) | 1 (4.0%) | >0.999 |
| amiodarone | 181 (36%) | 174 (37%) | 7 (28%) | 0.365 |
| AF ablation energy |  |  |  | 0.230 |
| cryoballoon | 65 (13%) | 64 (14%) | 1 (4.0%) |  |
| radiofrequency | 431 (87%) | 407 (86%) | 24 (96%) |  |
| AF ablation lesion set |  |  |  | 0.160 |
| PVI only | 294 (59%) | 284 (60%) | 10 (40%) |  |
| PVI+CAFE | 70 (14%) | 65 (14%) | 5 (20%) |  |
| PVI+line+CAFE | 18 (3.6%) | 17 (3.6%) | 1 (4.0%) |  |
| PVI+lines | 114 (23%) | 105 (22%) | 9 (36%) |  |
| Results are expressed as mean±SD or number (%). Abbreviations: ACEI: angiotensin-converting enzyme inhibitor; AF: atrial fibrillation; CFAE: complex fractionated atrial electrograms; PVI: pulmonary vein isolation; NOAC: novel oral anticoagulants. | | | | |
